# Structure-Based Optimization of One Neutralizing Antibody against SARS-CoV-2 Variants Bearing the L452R Mutation

**DOI:** 10.3390/v16040566

**Published:** 2024-04-05

**Authors:** Yamin Chen, Jialu Zha, Shiqi Xu, Jiang Shao, Xiaoshan Liu, Dianfan Li, Xiaoming Zhang

**Affiliations:** 1 Suzhou Medical College, Soochow University, Suzhou 215123, China; c783983640@163.com (Y.C.); liuxsh63@163.com (X.L.); 2 Key Laboratory of Immune Response and Immunotherapy, Shanghai Institute of Immunity and Infection, Chinese Academy of Sciences, Shanghai 200031, China; xushiqi@simm.ac.cn (S.X.); jshao@siii.cas.cn (J.S.); 3 CAS Center for Excellence in Molecular Cell Science, Shanghai Institute of Biochemistry and Cell Biology, Chinese Academy of Sciences, Shanghai 200031, China; zhajialu6@sibcb.ac.cn; 4 The CAS Key Laboratory of Receptor Research and State Key Laboratory of Drug Research, Shanghai Institute of Materia Medica, Chinese Academy of Sciences, Shanghai 201210, China; 5 Shanghai Sci-Tech Inno Center for Infection & Immunity, Shanghai 200052, China

**Keywords:** COVID-19, neutralizing antibody, structure-based antibody optimization, L452R mutation

## Abstract

Neutralizing antibodies (nAbs) play an important role against SARS-CoV-2 infections. Previously, we have reported one potent receptor binding domain (RBD)-binding nAb Ab08 against the SARS-CoV-2 prototype and a panel of variants, but Ab08 showed much less efficacy against the variants harboring the L452R mutation. To overcome the antibody escape caused by the L452R mutation, we generated several structure-based Ab08 derivatives. One derivative, Ab08-K99E, displayed the mostly enhanced neutralizing potency against the Delta pseudovirus bearing the L452R mutation compared to the Ab08 and other derivatives. Ab08-K99E also showed improved neutralizing effects against the prototype, Omicron BA.1, and Omicron BA.4/5 pseudoviruses. In addition, compared to the original Ab08, Ab08-K99E exhibited high binding properties and affinities to the RBDs of the prototype, Delta, and Omicron BA.4/5 variants. Altogether, our findings report an optimized nAb, Ab08-K99E, against SARS-CoV-2 variants and demonstrate structure-based optimization as an effective way for antibody development against pathogens.

## 1. Introduction

The currently circulating coronavirus disease 2019 (COVID-19) is caused by severe acute respiratory syndrome coronavirus 2 (SARS-CoV-2) [1,2]. The COVID-19 pandemic continues to spread more than three years after its emergence. The viral genome of SARS-CoV-2 encodes four structural proteins, including spike (S), membrane (M), envelope (E), and nucleocapsid (N) proteins [3]. The S protein is divided into two parts, the S1 subunit, which binds to host receptors through the receptor binding domain (RBD), and the S2 subunit, which mainly mediates the membrane fusion process between the virus and host cells [4,5].

As SARS-CoV-2 has spread, mutations in the S protein have continued to occur. It has been statistically shown that the circulating SARS-CoV-2 lineage is undergoing mutations in the nucleotides of the viral genome at a rate of one to two times per month [6]. Neutralizing antibodies (nAbs) are key to preventing and treating COVID-19, some of which have been urgently utilized as therapeutic drugs [7]. nAbs have been divided into seven categories based on the epitopes that bind RBDs [8]. Recently, RBD-targeting nAbs have been identified by several groups [9,10,11,12,13,14]. However, almost all emergency-use-authorization antibody-based drugs have partially lost efficacy or are ineffective with the emergence of new immune escape SARS-CoV-2 variants, particularly Omicron and its variants [15,16,17,18].

The structure-based optimization of antibodies is a useful strategy to improve currently available antibodies via computational virtual mutation (VM) and molecular dynamics (MD) simulations [19,20]. The increased binding capacity between the mutant antibodies and the RBD is mainly due to the increase in the electrostatic interactions and the free energy of solvation of the polar species. Additionally, using ADAPT (Assisted Design for Antibody and Protein Therapeutics), a platform that combines structure-based computational predictions with experimental testing, the binding affinity can be further optimized [21,22].

Previously, we have cloned one potent RBD-binding nAb Ab08 from one convalesced patient following SARS-CoV-2 infection. Ab08 neutralized the prototype and several variants, including Omicron BA.1, but showed decreased efficacy against the variants harboring the L452R mutation [23]. In this study, to overcome the effect by the L452R mutation, we performed the structure analysis and designed several Ab08 derivatives. We screened and validated one derivative, Ab08-K99E, which exhibited much increased neutralizing capacity against pseudotyped SARS-CoV-2 Delta and Omicron BA.4/5 variants compared to the original Ab08.

## 2. Materials and Methods

### 2.1. Cell Culture

HEK 293T and human ACE2-overexpressing HEK 293T cells (293T-hACE2) were cultured at 37 °C in Dulbecco’s Modified Eagle’ Medium (DMEM) high glucose (Corning, Corning, NY, USA) supplemented with 10% fetal bovine serum (FBS, Gibco, Waltham, MA, USA) and 1% penicillin/streptomycin (P/S). The 293T-hACE2 cell line was constructed previously [24,25]. ExpiCHO-S^TM^ cells (Thermo Fisher, Waltham, MA, USA) were grown in ExpiCHO expression medium (Gibco).

### 2.2. Antibody Production and Purification

Heavy-chain plasmids were synthesized by Genscript. Light-chain plasmids were generated as previously described [23]. IgG1 heavy-chain and IgL light-chain plasmids were transfected into cells at a ratio of 1:2. The test expression of each isolated antibody was carried out in 0.5 mL cultures of HEK 293T cells in 24-well plates for 3 days. Cell supernatants were clarified and used in neutralization screens. Antibodies of interest were produced in ExpiCHO-S cells by using a transfection reagent according to the manufacture’s procedure (Cat. A29133, Thermo Fisher). The supernatants were harvested after 9 days of culture and the antibodies were purified using protein G agarose resin 4FF (Yeasen, Shanghai, China) according to the manufacturer’s protocol.

### 2.3. Pseudovirus Neutralization Assay

HEK 293T cells grown in 10 cm dish were co-transfected using PEI (polysciences) with 10 μg of MLV Gag-Pol packaging plasmid, 10 μg of transfer plasmid containing a luciferase reporter gene, and 2 μg of plasmids encoding SARS-CoV-2 S protein. The cells were incubated with the transfection mixture for 6 h. After washing once with DMEM, fresh DMEM medium supplemented with 10% FBS was added and incubated at 37 °C for 48 h. The culture supernatant was harvested and centrifugated. Then, the pseudovirus was stored at −80 °C until use.

The pseudovirus neutralization assays were performed using 293T-hACE2 cell lines. Before the experiment, 293T-hACE2 cells were seeded into 96-well plates and incubated overnight. Various concentrations of nAbs (4-fold serial dilution using DMEM, 45 μL) were mixed with the 90 μL of SARS-CoV-2 pseudovirus in 96 well-plates. The mixture was incubated for 1 h at 37 °C, supplied with 5% CO_2_, and then added to the plates. After 12 h, the mixtures were removed and the cells were followed by the addition of fresh 10% FBS DMEM. At 48 h post infection, the luciferase luminescence (RLU) of each well was measured with the luciferase assay system (Promega, Fitchburg, WI, USA). The inhibition rates of each concentration of the sample were calculated according to the luminescence values as [1 − (RLU of sample − RLU of the cell-only control)/(RLU of the virus-only control − RLU of the cell-only control)] × 100%.

### 2.4. Binding ELISA Assay

To determine the binding activities of SARS-CoV-2 RBD proteins with antibodies, SARS-CoV-2 RBD proteins were coated in ELISA 96-well plates (Nunc) with 100 ng/well at 4 °C overnight. Then, the plates were blocked with 5% Newborn Calf Serum (NBCS) in PBS at 37 °C for 1 h. Antibodies were 4-fold diluted and added into plates at room temperature for 1 h, followed by incubation with horseradish peroxidase (HRP)-conjugated anti-human IgG (Sourthern biotech, diluted 1:8000, Birmingham, AL, USA) for 0.5 h at room temperature. After washing and color development, absorbance was monitored at 450 nm.

### 2.5. Surface Plasmon Resonance

Binding kinetics measurements of SARS-CoV-2 antibodies to prototype, Delta, and Omicron BA.4/5 RBD proteins were performed using a Biacore T200 instrument (Cytiva, Marlborough, MA, USA) in HBS-EP + 1 × running buffer. The RBD proteins were immobilized on a Sensor Chip CM5 (Cytiva), and antibodies were injected over the two flow cells at a range of five concentrations prepared by serial 2-fold dilutions, at a flow rate of 30 μL/min. The response was recorded using Biacore Insight Evaluation Software (3.2.1) at 25 °C, and the resulting data were analyzed by the Software ware using the 1:1 binding model.

The RBD for the prototype strain was synthesized by AtaGenix. The RBDs for other variants were purchased from Sino Biological (Delta RBD, Cat. 40592-V08H90; Omicron BA.4/5 RBD, Cat. 40592-V08H130, Beijing, China).

### 2.6. Structure Visualization and Analysis

Structures were visualized and analyzed using Pymol (2.3.3) and Coot (0.9) [26]. Virtual mutations were performed using Coot and structural figures were generated using Pymol (2.3.3).

## 3. Statistical Analysis

GraphPad Prism software (version 9.4.1) was used for the statistical analysis of data and graph generation. Data are presented as mean ± SEM. For two-group comparison, the unpaired *t*-test was utilized. Nonlinear regression was analyzed using Variable Slope (Four Parameters).

## 4. Results

### 4.1. Structure-Based Design of Ab08 Derivatives for Binding with L452R

The L452R mutation of SARS-CoV-2 is notorious in escaping numerous neutralizing antibodies, including R1-32 [27], FC08, BD-380, BD-421, BD-901, C091, and XGv-271 [28]. This list also includes Ab08, a human monoclonal antibody isolated from patients infected with the original strain in early 2020 [23]. This escaping effect compromises Ab08’s further development potential despite its excellent therapeutic efficacy in SARS-CoV-2-infected mice.

Notably, the molecular interaction detail between Ab08 and the RBD has been elucidated through crystallography in our previous study. This high-resolution structural information could be harnessed to rationally design antibody derivatives to enhance affinity, as demonstrated in a similar study of ours [29]. Thus, we performed virtual mutagenesis around the interaction interface.

**Figure 1:**
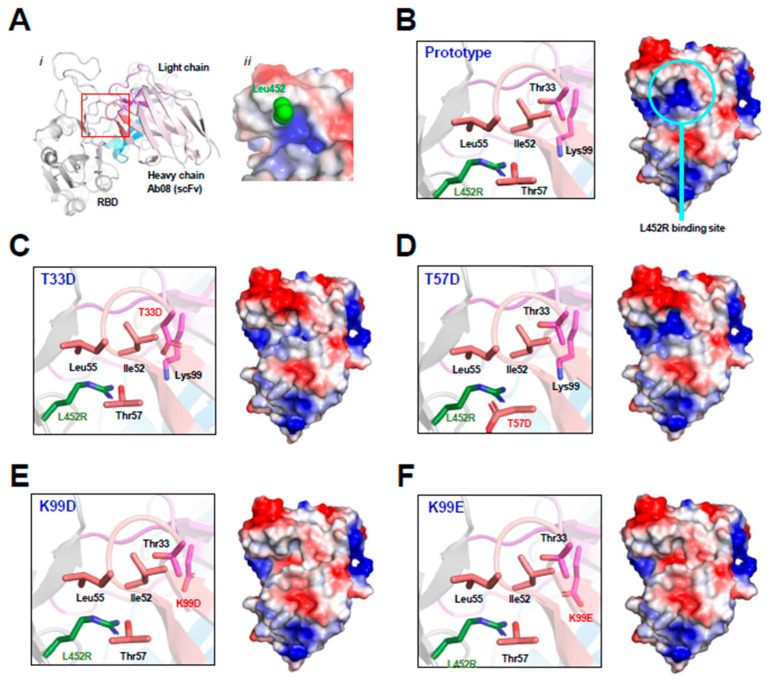
Structure-based design of Ab08 derivatives. (**A**) Overview ((**i**), cartoon) and expanded view ((**ii**), electropotential surface) of the Ab08-RBD structure. Various parts, including the side chain of Leu453 (green sphere), are appropriately shown and labeled. (**B**–**F**) Expanded view of the L452R-binding site (cartoon and ribbon, left) and electropotential surface of Ab08 (right) of Ab08. In electropotential representations, blue and red indicates positively and negatively charged surfaces, respectively.

As shown by the Ab08-RBD complex structure (Figure 1A), the RBD L452R mutation occurs at the binding interface near the CDR2 and CDR3 regions of the heavy chain of Ab08. The Ab08 region interacting with Leu452 in the prototype RBD exhibits a positively charged pocket (Figure 1A,B). The arginine side chain from the L452R mutant would weaken the Ab08-RBD interactions owing to electrorepulsion with this pocket. To circumvent this charge–charge repulsion, we designed virtual mutations around this pocket by introducing negatively charged residues. They include T33D (Figure 1C), T57D (Figure 1D), K99D (Figure 1E), and K99E (Figure 1F). As judged by the electropotential surface, K99D and K99E reversed the charge of the putative L452R-binding pocket. Therefore, these mutations were expected to increase affinity with RBD owing to the electrostatic interactions between the positively charged Arg452 from RBD and the negatively charged residues at residues 33/99 of Ab08.

### 4.2. Ab08-K99E Effectively Neutralizes Pseudotyped SARS-CoV-2 Delta and Omicron BA.4/5 Variants

**Figure 2:**
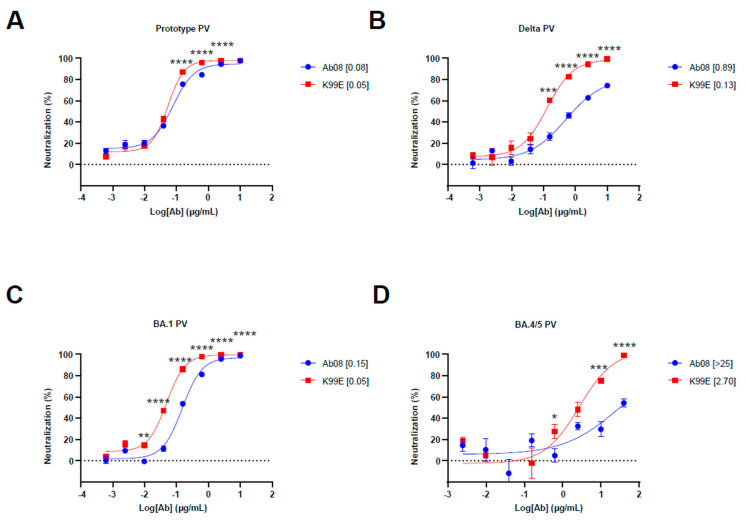
Neutralization activity of Ab08 and Ab08-K99E. (**A**–**D**) Neutralization assays of Ab08 and Ab08-K99E using pseudoviruses from SARS-CoV-2 prototype (**A**), Delta (**B**), and Omicron variants, as indicated (**C**,**D**). Data are plotted as mean ± SEM of four replicate wells. Numbers in brackets are IC_50_ values in μg/mL. *p* values were analyzed with an unpaired *t*-test and indicated as follows: * *p* < 0.05; ** *p* < 0.01; *** *p* < 0.001; **** *p* < 0.0001.

To test the structure-based derivatives, we synthesized the antibody heavy and light chain genes of Ab08 derivatives and inserted them into the expression plasmids. We first screened the neutralizing effects of the supernatants from the above plasmid-transfected HEK 293T cells by using the prototype, Delta, and Omicron BA.1 pseudoviruses. We found that K99D, K99E, T33D, and T57D derivatives were effective in neutralizing the Delta pseudovirus, which harbored the L452R mutation (Figure S1A). We then used quantitative assays to measure the half maximal inhibitory concentration (IC_50_) of these nAbs as well as the original Ab08 (Figure 2A–D and Figure S1B). The results showed that the Ab08-K99E had low IC_50_ for the Delta pseudovirus (IC_50_ = 0.13 μg/mL) and the Omicron BA.4/5 pseudovirus (IC_50_ = 2.70 μg/mL). The Ab08-K99E also showed better neutralizing effects for the prototype and Omicron BA.1 pseudoviruses in comparison to the other nAbs (Figure 2A–D and Figure S1B). Collectively, we have generated a structure-guided antibody Ab08-K99E with improved neutralizing efficacy to overcome the L452R mutation prevalent in new SARS-CoV-2 variants.

### 4.3. Ab08-K99E Displays Higher Affinity for RBDs from SARS-CoV-2 Delta and Omicron BA.4/5 Variants

**Figure 3:**
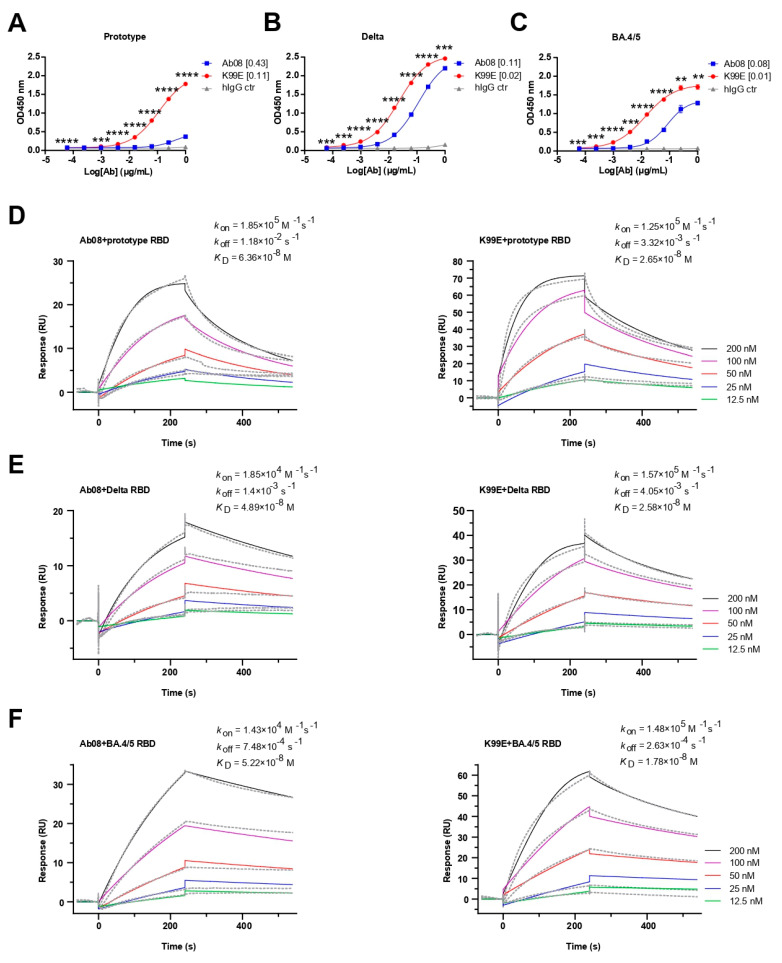
Binding activities and kinetics of Ab08 and Ab08-K99E. (**A**–**C**) Reactivities of Ab08 and Ab08-K99E to the SARS-CoV-2 RBDs measured via ELISA. Data are mean ± SEM of triplicate wells. Numbers in brackets are EC_50_ values in μg/mL. Comparisons between Ab08 and Ab08-K99E were performed using an unpaired *t*-test. *p* values were indicated as follows: ** *p* < 0.01; *** *p* < 0.001; **** *p* < 0.0001. hIgG synthesized by AtaGenix served as IgG1 isotype control (hIgG ctr). (**D**–**F**) Binding affinities of Ab08 and Ab08-K99E to SARS-CoV-2 prototype and variant RBDs using surface plasmon resonance (SPR). Gray dashed lines indicate experimental data and solid colored lines indicate fitted curves. One representative experiment out of three is shown.

Next, we compared the binding property of the purified Ab08-K99E and Ab08 to the RBDs from the SARS-CoV-2 prototype, Delta, and Omicron BA.4/5 variants via ELISA. Both neutralizing antibodies were able to bind the RBDs in a dose-dependent manner, with Ab08-K99E binding to the prototype RBD at a half maximal effective concentration (EC_50_) of 0.11 μg/mL (Figure 3A), the Delta RBD at an EC_50_ of 0.02 μg/mL (Figure 3B), and the Omicron BA.4/5 RBD at an EC_50_ of 0.01 μg/mL (Figure 3C). By comparison, the EC_50_ values for the RBDs of the prototype, Delta, and Omicron BA.4/5 by Ab08 were 0.43 μg/mL, 0.11 μg/mL, and 0.08 μg/mL, respectively (Figure 3A–C).

The binding affinities of the two antibodies to different RBDs were further determined using surface plasmon resonance (SPR). Ab08-K99E showed high binding affinities to the RBDs from the prototype (*K*_D_ = 2.65 × 10^−8^ M), Delta (*K*_D_ = 2.58 × 10^−8^ M), and Omicron BA.4/5 (*K*_D_ = 1.78 × 10^−8^ M) compared to those of Ab08 (the prototype: *K*_D_ = 6.36 × 10^−8^ M; Delta: *K*_D_ = 4.89 × 10^−8^ M; and Omicron BA.4/5: *K*_D_ = 5.22 × 10^−8^ M) (Figure 3D–F). In addition, Ab08-K99D and Ab08-T33D also showed high binding affinities for these RBDs (Figure S2). Collectively, the above results suggest that the structure-based optimization of Ab08-K99E increases the binding affinities to the prototype and Delta and Omicron BA.4/5 RBDs which are likely responsible for the enhanced neutralizing efficacy.

## 5. Discussion

The Omicron variants have attracted great attention due to their exceptional ability to evade immune response and their high transmissibility [30]. Therefore, there is a need for the development of neutralizing antibodies with broad protection against a wide range of Omicron and other potential variants. In our previous study [23], we isolated and characterized one nAb Ab08 that neutralized the SARS-CoV-2 prototype and a panel of variants, including Omicron BA.1. However, it was less effective against Delta and Omicron BA.4/5 variants which bear the L452R mutation. We found that Leu452 was located on the epitope of Ab08, as shown in the crystal structure [23]. To improve the efficacy of Ab08, we used computational biology to design several Ab08 derivatives and tried to overcome the negative effect exerted by the L452R mutation.

Among the new antibodies, we found that Ab08-K99E was particularly effective in neutralizing the pseudotyped SARS-CoV-2 Delta and Omicron BA.4/5 variants which have the L452R mutation. In addition, Ab08-K99E also had an increased neutralizing effect on the prototype and Omicron BA.1 variant compared to the original Ab08. The underlying mechanism could be ascribed to the enhanced affinities between the antibody and the target epitope, because of the electrostatic interactions between the positively charged Arg452 from RBD and the negatively charged residue introduced at residue 99 in Ab08-K99E. Indeed, in our previous study, a rationally designed nanobody exhibited ~2-fold increase in binding affinity and nearly 30-fold increase in neutralizing efficiency [29].

Structure-based antibody optimization has attracted substantial attention in the field of antibody development, mostly due to rapid advances in antigen–antibody complex structure analyses, antibody modeling and prediction with increasing accuracy, and deep learning. By applying computational virtual mutation and functional screening, the binding affinity of the CR3022 antibody to SARS-CoV-2 RBDs was significantly enhanced more than ten times after the introduction of the S103F/Y mutation in HCDR-3 and the S33R mutation in LCDR-1 [20]. With the aid of RosettaAntibodyDesign (RAbD) [31], Yang et al. established an integrated structure-based antibody design framework. The neutralizing activity of a panel of derivatives from S2H97 (also a SARS-CoV-2 RBD-targeting antibody) against multiple SARS-CoV-2 variants was increased from several to hundreds of times [32]. Moreover, Shan et al. utilized a geometric deep learning algorithm to optimize the RBD-targeting antibody P36-5D2’s sequences with improved potency by 10- to 600-fold against SARS-CoV-2 variants [33]. Furthermore, a language-model-guided affinity maturation algorithm was successfully used to optimize the neutralizing antibodies against influenza virus and ebolavirus [34]. Our work joins these studies as an encouraging case for structure-based antibody engineering, providing a promising strategy to modify current antibodies against future escaping variants.

In short, the current study reinforces the idea that the structure-based optimization of already available antibodies is an effective way for therapeutic and prophylactic antibody improvement, to combat COVID-19 and other infectious diseases.

## Data Availability

All data generated or analyzed during this study are included in this published article.

## Supplementary Materials

The following supporting information can be downloaded at: https://www.mdpi.com/article/10.3390/v16040566/s1, Supplementary Figure S1. Identification of Ab08-K99E from pseudovirus neutralization assays; Supplementary Figure S2. Binding kinetics of Ab08-K99D and Ab08-T33D.
