# Supplementary figures and images for: Structure-Based Optimization of One Neutralizing Antibody against SARS-CoV-2 Variants Bearing the L452R Mutation
Source: Viruses. 2024 Apr 5;16(4):566. doi: 10.3390/v16040566 (PMC11053997; doi:10.3390/v16040566)

# A

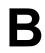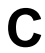[illegible]

Supplement: Supplementary file 1 [file viruses-16-00566-s001.zip › Supplementary files/Figure S1.pdf]

# Supplementary Figure 2

A

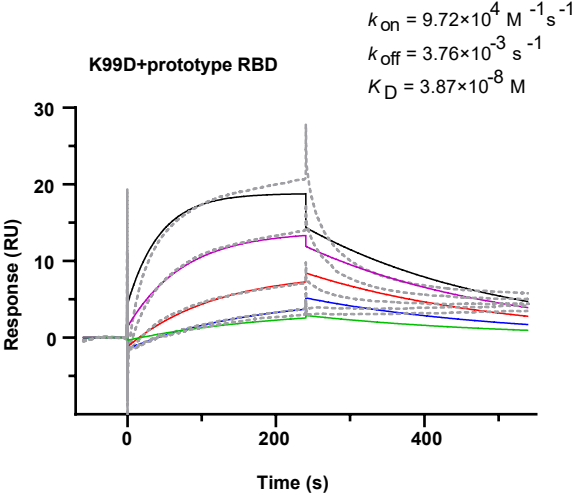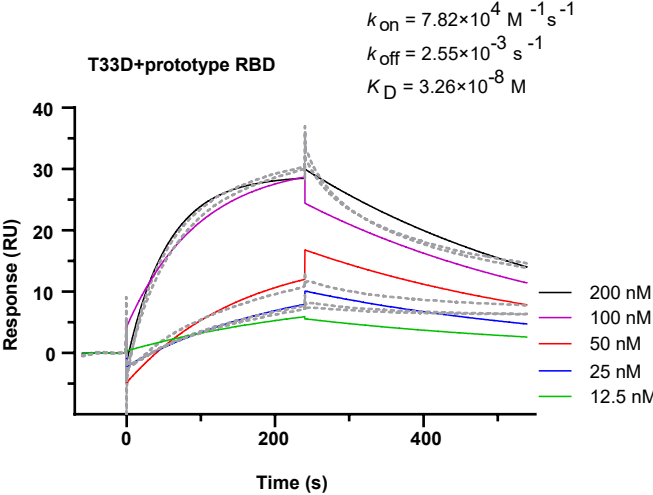

B

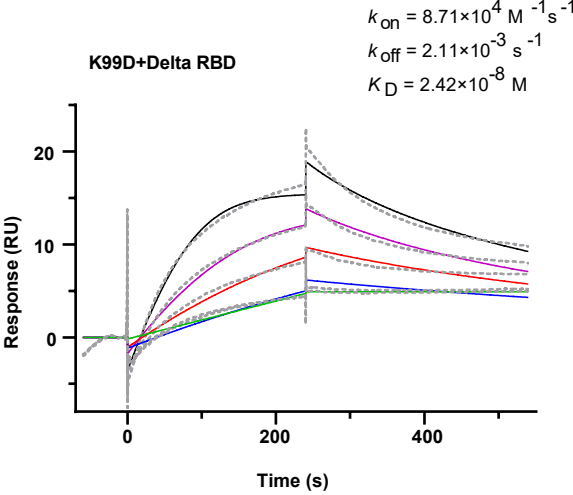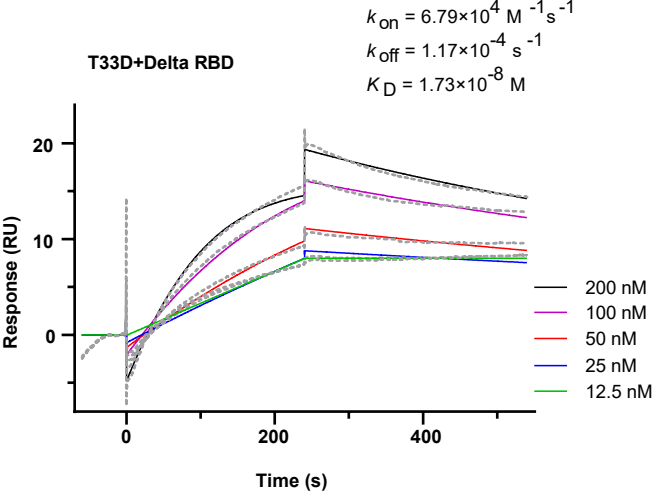

C

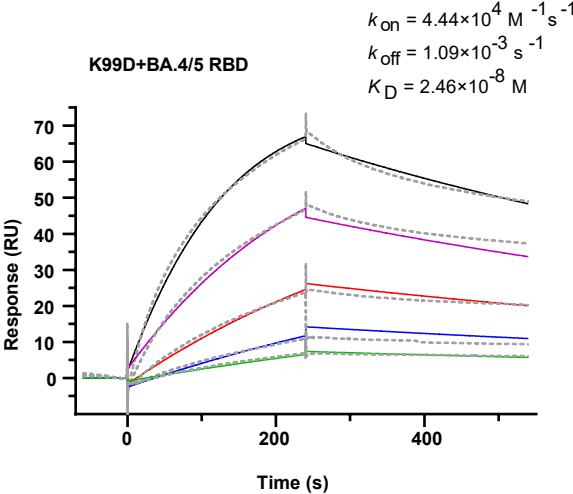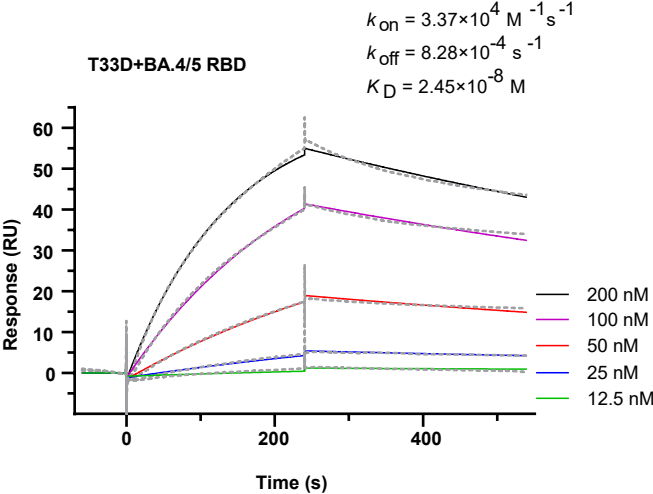

Supplement: Supplementary file 1 [file viruses-16-00566-s001.zip › Supplementary files/Figure S2.pdf]
